# Molecular characterization of non-aureus staphylococci and *Mammaliicoccus* from Hipposideros bats in Southwest Nigeria

**DOI:** 10.1038/s41598-024-57190-z

**Published:** 2024-03-22

**Authors:** Tomiwa O. Adesoji, Uwem E. George, Taofiq A. Sulayman, Jessica N. Uwanibe, Idowu B. Olawoye, Joseph O. Igbokwe, Tobi G. Olanipekun, Richard A. Adeleke, Akintayo I. Akindoyin, Temitope J. Famakinwa, Andrew M. Adamu, Christabel A. Terkuma, Grace O. Ezekiel, Philomena E. Eromon, Anise N. Happi, Taiwo O. Fadare, Adebayo O. Shittu, Christian T. Happi

**Affiliations:** 1 Department of Microbiology, Obafemi Awolowo University, Ile-Ife, Osun State Nigeria; 2 Department of Biological Sciences, Faculty of Natural Sciences, Redeemer’s University, Ede, Osun State Nigeria; 3 African Centre of Excellence for Genomics of Infectious Diseases, Redeemer’s University, Ede, Osun State Nigeria; 4 Department of Zoology, Obafemi Awolowo University, Ile-Ife, Osun State Nigeria; 5 Department of Veterinary Microbiology, University of Ibadan, Ibadan, Oyo State Nigeria; 6 Immunology and Infectious Diseases, College of Veterinary Medicine, Cornell University, New York, NY 14853 USA; 7 Institute of Ecology, Obafemi Awolowo University, Ile-Ife, Osun State Nigeria; 8 Natural History Museum, Obafemi Awolowo University, Ile-Ife, Osun State Nigeria; 9 Department of Veterinary Public Health and Preventive Medicine, Faculty of Veterinary Medicine, University of Abuja, Federal Capital Territory, Abuja, 900105 Nigeria; 10 Australian Institute of Tropical Health and Medicine, Division of Tropical Health and Medicine, James Cook University, Townsville, QLD 4811 Australia; 11 College of Public Health, Medical and Veterinary Sciences, James Cook University, 1 James Cook Drive, Bebegu Yumba Campus, Douglas, QLD 4811 Australia

**Keywords:** Microbiology, Molecular biology, Diseases, Medical research

## Abstract

Bats are not only ecologically valuable mammals but also reservoirs of zoonotic pathogens. Their vast population, ability to fly, and inhabit diverse ecological niches could play some role in the spread of antibiotic resistance. This study investigated non-aureus staphylococci and *Mammaliicoccus* colonization in the *Hipposideros* bats at Obafemi Awolowo University, Ile-Ife, Nigeria. Pharyngeal samples (*n* = 23) of the insectivorous bats were analyzed, and the presumptive non-aureus staphylococcal and *Mammaliicoccus* isolates were confirmed by matrix-assisted laser desorption ionization-time of flight mass spectrometry (MALDI-TOF MS). The isolates were characterized based on antibiotic susceptibility testing and whole-genome sequencing (WGS). Six bacterial genomes were assembled, and three species were identified, including *Mammaliicoccus sciuri* (*n* = 4), *Staphylococcus gallinarum* (*n* = 1), and *Staphylococcus nepalensis* (*n* = 1). All the isolates were resistant to clindamycin, while the *M. sciuri* and *S. gallinarum* isolates were also resistant to fusidic acid. WGS analysis revealed that the *M. sciuri* and *S. gallinarum* isolates were *mecA*-positive. In addition, the *M. sciuri* isolates possessed some virulence (*icaA, icaB, icaC,* and *sspA*) genes. Multi-locus sequence typing identified two new *M. sciuri* sequence types (STs) 233 and ST234. The identification of these new STs in a migratory mammal deserves close monitoring because previously known ST57, ST60, and ST65 sharing ack (8), ftsZ (13), glpK (14), gmk (6), and tpiA (10) alleles with ST233 and ST234 have been linked to mastitis in animals. Moreover, the broad host range of *M. sciuri* could facilitate the dispersal of antibiotic resistance genes. This study provides evidence of the importance of including migratory animals in monitoring the development and spread of antibiotic resistance.

## Introduction

Bats are one of the most diverse animal groups, with over 1,300 species living in different habitats and climatic zones^1^. They are classified into two main groups, i.e., insectivorous (insect-eaters) and fructivorous (fruit-eaters). The Old-World leaf-nosed bats, also known as *Hipposideridae* (insectivorous bats), have been identified in tropical and subtropical regions of Africa, the Middle East, Asia, and Australia. Bats are vital pollinators of commercially essential plants and animal protein sources^2–4^. On the other hand, bats have also been recognized as potential reservoirs and vectors of zoonotic pathogens^5–8^.

The *Staphylococcaceae* family comprises nine genera, including *Abyssicoccus*, *Aliicoccus*, *Corticicoccus*, *Jeotgalicoccus*, *Nosocomiicoccus*, *Salinicoccus*, *Macrococcus*, *Staphylococcus*, and the recently classified *Mammaliicoccus*. The genus *Mammaliicoccus* consists of five members earlier classified as the *Staphylococcus sciuri* group (*S. sciuri*, *S. lentus*, *S. vitulinus*, *S. stepanovicii* and *S. fleurettii*)^9^. The genera *Staphylococcus* and *Mammaliicoccus* colonize animal and human hosts^10–12^. The non-aureus staphylococci and mammaliicocci have gained public health attention as they have been implicated in mild to life-threatening infections, including skin and soft tissue infections and neonatal sepsis in humans^13,14^. In addition, they possess some antibiotic-resistance genes^15^. These include the *erm* gene encoding resistance to the macrolide/lincosamide/streptogramin B (MLS_B_)^16^, *salA* for lincosamide/streptogramin A^17^, and the *mecA-mecC* hybrid SCC*mec* element encoding the beta-lactam resistance^18^. Furthermore, it is postulated that *M. sciuri* is a reservoir of antimicrobial resistance gene determinants and transferred to other virulent members of the *Staphylococcaceae* family, particularly *S. aureus*^11,19^.

The “One-Health” concept has demonstrated the need to include wild animals in antimicrobial resistance studies as they could serve as important vehicles driving the development and dissemination of antibiotic resistance determinants. Previous studies on bacterial colonization in bats have suggested that these migratory mammals are colonized by some medically important bacteria including staphylococci^20,21^. In Nigeria, bat roosting sites often overlap with areas of human occupation and habitation and thus increase the chance of cross-species transmission of bat pathogens that may cause zoonotic infections in the country. The occurrence of members of the *S. aureus* complex from the faecal samples of fructivorous bats (*E. helvum*) has been previously reported in Nigeria^22^. However, these studies have not investigated the occurrence of non-aureus staphylococci (NAS) in these arboreal mammals, especially in insectivorous bats. In addition, there is a paucity of data on the molecular characterization of NAS and *Mammaliicoccus* from insectivorous bats in Nigeria. Therefore, this study seeks to investigate the occurrence and characterize NAS and mammaliicocci from *Hipposideros* bats in Ile-Ife, Nigeria.

## Results

### Identification of isolates in the Staphylococcaceae family and antibiotic susceptibility testing

Pharyngeal samples (n = 23) were obtained, and the sequencing of the MT-Cytb gene confirmed the bats as *Hipposideros larvatus* in the *Hipposideridae* family. Nine staphylococcal isolates were recovered, of which six were randomly selected for identification using the matrix-assisted laser desorption/ionisation-time of flight mass spectrometry (MALDI-TOF MS—Bruker, Dalton, Germany) and whole genome sequencing (WGS). The isolates comprised *Mamaliicoccus sciuri* (n = 4), *S. gallinarum* (n = 1), and *S. nepalensis* (n = 1). The antibiotic susceptibility testing result showed that all the isolates were resistant to clindamycin, and the *M. sciuri* and *S. gallinarum* isolates exhibited resistance to fusidic acid (Table 1). In addition, the isolates were susceptible to other antibiotics including cefoxitin, chloramphenicol, ciprofloxacin, erythromycin, penicillin G and gentamicin.

**Table 1:** Antibiotic resistance profile, resistance genes and virulence factors detected in isolates.

ID	Identity	Antibiotic resistance profile	Putative antibiotic resistance gene detected	Putative virulence factors detected
2a	*S. nepalensis*	CD		*atl, ebp, nuc, lip, sspA, capB, capC*
3a	*M. sciuri*	FD-CD	*mecA, SalA*	*icaA, icaB, icaC, sspA*
6a	*M. sciuri*	TM-CD-FD	*mecA, SalA, qacE*	*icaA, icaB, icaC, sspA*
9a	*M. sciuri*	TM-CD-FD	*mecA*	*clfB, icaA, icaB, icaC, sspA*
11a	*S. gallinarum*	TM-CD-FD	*mecA, qacD*	
35b	*M. sciuri*	TM-CD-FD	*mecA, SalA*	*icaA, icaB, icaC, sspA*

N/B: TM: trimethoprim; CD: clindamycin; FD: fusidic acid.

### Quality Control and Whole-genome sequencing

**Figure 1:**
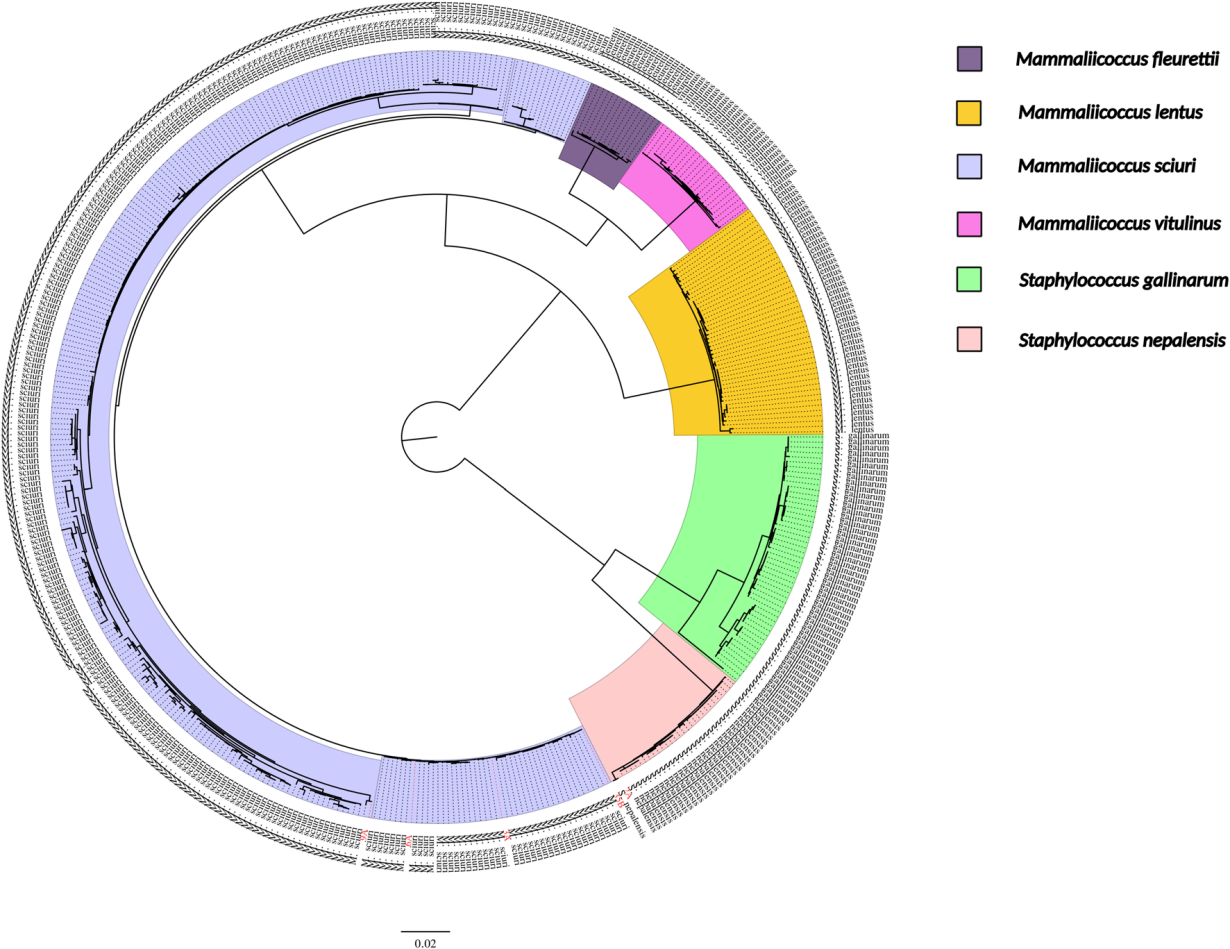
Maximum likelihood core genome phylogeny of *M. fleurettii, M. lentus, M. sciuri, M. vitulinus, S. gallinarum, and S. nepalensis using* 449 publicly available assemblies and five genome assemblies from this study indicated with red taxon labels (excluding the partial *S. gallinarum* genome). Clades are coloured according to species. The phylogenetic tree is constructed from a total of 5,395 SNP sites across the core genomes of 454 assemblies using an ultrafast bootstrap value of 1000.

The generated contigs for the isolates ranged between 61 and 712, with a GC content of 32.53 to 33.50% (Table 2). Phylogenetic analysis based on core genome SNPs revealed that the Nigerian isolates from *Hipposidero*s bats clustered alongside other global *M. sciuri *(Min.—Max.: 0—77 SNPs) and *S. nepalensis* (Min.—Max.: 0—27 SNPs) genomes, including isolates previously reported in humans (Fig. 1 and Supplementary Table 1).

**Table 2:** Genome statistics of assembled contigs.

Sample ID	Probable isolate	Total length (bp)	No. of contigs	N50	GC (%)
2A	*Staphylococcus nepalensis*	2,662,421	61	197,938	33.02
3A	*Mammaliicoccus sciuri*	2,543,062	542	7503	32.62
6A	*Mammaliicoccus sciuri*	2,563,326	468	9299	32.62
9A	*Mammaliicoccus sciuri*	2,490,609	668	6047	32.84
11A	*Staphylococcus gallinarum*	1,347,617	712	2361	33.50
35B	*Mammaliicoccus sciuri*	2,592,917	486	8993	32.53

Also, WGS revealed that the *M. sciuri* and *S. gallinarum* isolates were *mecA*-positive. Furthermore, the *M. sciuri* isolates possessed some virulence (*icaA, icaB, icaC,* and *sspA*) genes. Antibiotic resistance genes were not identified in *S. nepalensis*. However, virulence factors were detected, including autolysin (*atl*) and elastin-binding proteins (*ebp*) (Table 1).

### Multi-locus sequence typing

**Figure 2:**
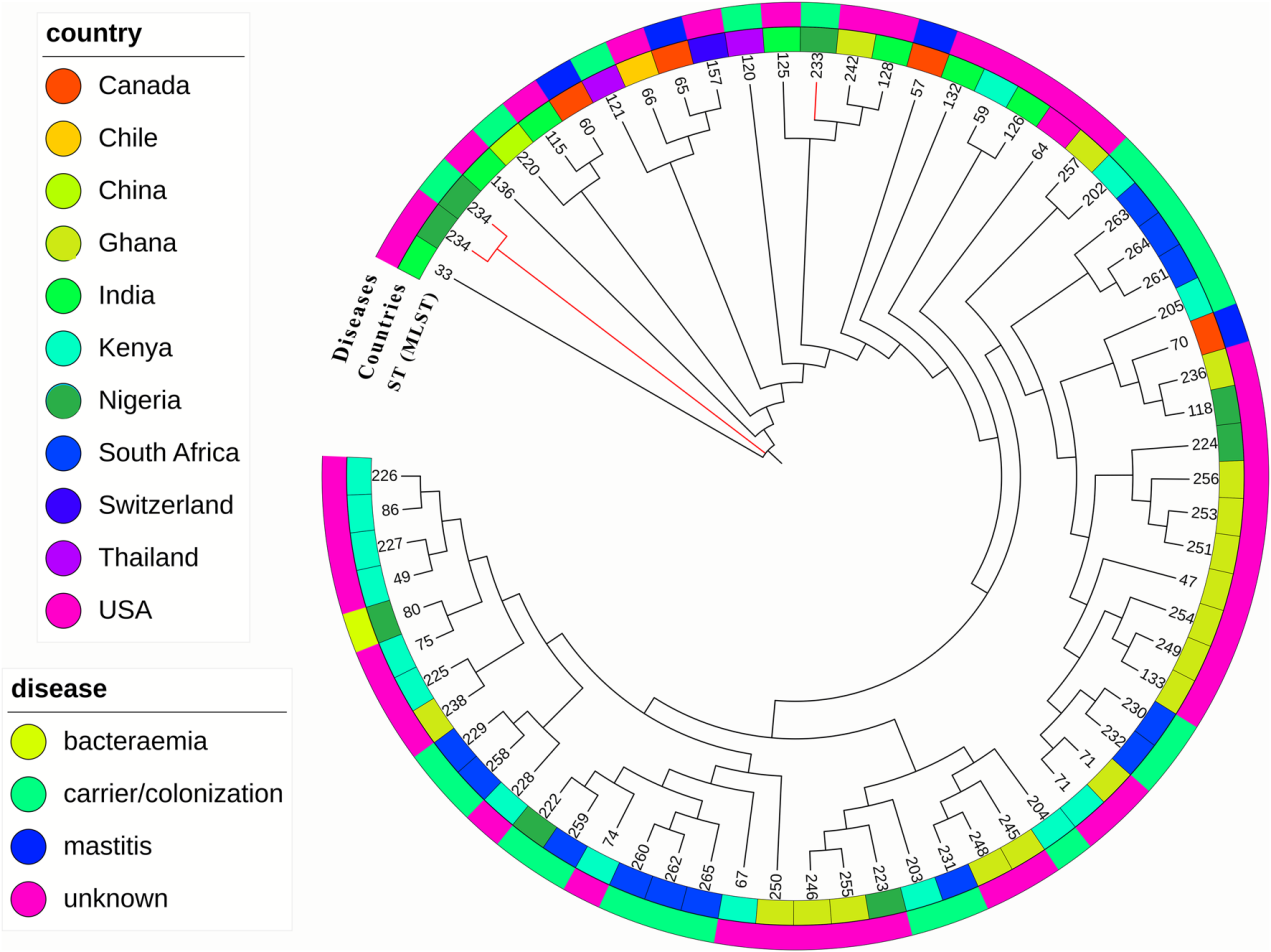
NJT obtained from the concatenated nucleotide sequences on the MLST database using the iTOL tool.

Of the four *M. sciuri* isolates, two new STs (ST233 and ST234) were identified, with a tree branch highlighted in red (Fig. 2). A Neighbour-joining tree (NJT) was constructed using the MLST concatenated nucleotide sequences of related *M. sciuri* ST types (at least four similar alleles) globally, including African STs in the database (accessed on 18 September 2023) (Fig. 2). *M. sciuri* ST233 and ST234 from *Hipposideros* bats in Nigeria were distinct from previously described STs in Nigeria but clustered (having 1 to 2 allelic differences) with STs from other animal species in Canada, Switzerland, India, and Thailand (Fig. 2 and Supplementary Table 2).

## Discussion

In this study, NAS, including *S. nepalensis* and *S. gallinarum*, were colonizers of *Hipposiderous* bats. *S. nepalensis* has been previously implicated in human and animal infections^23^. In addition, *S. nepalensis* has been reported in various environments and clinical fomites^24^. They have also been previously reported in Slovakia in *Myotis myotis* and *Rhinolophus hipposideros* (insectivorous bats) guano^20,25^. Various NAS have been reported to possess different antibiotic resistance and virulence genes, which could affect the treatment of infections caused by the pathogen in humans and animals^25^. Furthermore, these resistance genes could be transferred to more virulent members of the *Staphylococcaceae* family, including *S. aureus*^26^. In this study, antibiotic-resistance genes were not detected in the *S. nepalensis*. Nevertheless, some virulence factors were identified, including the autolysin (*atl*) and the elastin binding proteins (*ebp*). These genes with other virulence genes, including *nuc*, *sspA* and *lip* (present in the isolate), could facilitate adhesion to susceptible hosts and the subsequent establishment of infection^27^. This observation suggests that insectivorous bats should be considered a potential source of pathogenic bacteria with zoonotic potential. This observation further reiterates the public health concern associated with human co-habitation with bats (both fructivorous and insectivorous) as previous studies in Nigeria have focused on fructivorous bats (*E. helvum*) and *S. aureus*.

Genomic phylogeny using core genomes of NAS and mammaliicocci isolated in the study with other global isolates from humans showed a minimal divergence between the bacterial isolates from human and bat hosts. This observation suggests that there is a possibility of zoonosis/reverse zoonosis leading to the exchange of antimicrobial resistance and virulence genes, just as methicillin-resistant *Staphylococcus aureus* (MRSA) has been reported in livestock and companion animals^28,29^.

*Mammaliicoccus* species, formerly regarded as contaminants in the clinical diagnosis of infections, are now of public health attention. They have been implicated in both human and animal infections ranging from skin and soft tissue infections^30^, neonatal sepsis^31^ in humans, pneumonia and bovine mastitis^32^ in animals. In addition to their pathogenic potentials, they have been reported to be carriers of several antibiotic-resistance genes, including the methicillin resistance genes^12,33^. In this study, *M. sciuri* was recovered from migratory mammals. These isolates were susceptible (phenotypically) to some antibiotics. However, they possessed the corresponding antibiotic-resistance genes. For instance, the *mecA* gene was detected in all *M. sciuri* but conversely showed susceptibility to cefoxitin (a surrogate marker for methicillin resistance). In addition, no SCC*mec* element was detected in these isolates using the SCC*mec*Finder. The original *mecA* gene was located on the chromosome and it is postulated to have originated from these species^34^. These isolates also lacked other antibiotic resistance genes, leading to the observed susceptibility to cefoxitin and other antibiotics^35^.

In addition, 75% (3/4) of the *M. sciuri* isolates were *salA*-positive, the genetic basis for clindamycin resistance. Also, it confers moderate lincosamide resistance (eight times the MIC of lincomycin) and high-level (64 times the MIC) streptogramin resistance^17^. This gene has been reported to be intrinsically present in *M. sciuri* and is located between two housekeeping genes in the core genome of *M. sciuri*^17^.

Interestingly, we observed that one of the *M. sciuri* isolates was *qacE*-positive. The gene encodes multidrug efflux pumps and confers resistance to the quaternary ammonium compounds and intercalating agents^36,37^. Several *qac* genes have been reported in staphylococci^38^. However, the *qacE* gene is observed mainly in Gram-negative bacteria, especially *Enterobacteriaceae* and *Pseudomonas aeruginosa*^39^. It is associated with a mobile genetic element (MGE), class 1 integrons in Gram-negative bacteria^40^. This MGE has been previously investigated and reported in various staphylococcal species^41–43^ and could be disseminated among isolates by horizontal gene transfer^44^. Although the *qacE* gene observed in the *M. sciuri* in this study is chromosomal, the presence of this gene in isolates from migratory mammals such as bats could be a public health concern as it could be transferred to more pathogenic staphylococci, including *S. aureus*, via horizontal gene transfer. This trend underscores the need for continuous monitoring of wildlife for the dissemination of antibiotic-resistant bacteria.

The *M. sciuri* isolates exhibited phenotypic resistance to trimethoprim and fusidic acid without the corresponding antibiotic resistance genes. Trimethoprim resistance is conferred by various *dfr* genes among the staphylococci^45^. However, these genes were undetected in these isolates. It is plausible that these isolates carry an alternative trimethoprim resistance determinant that is unavailable in the databases. Furthermore, the different *fus* genes that encode fusidic acid resistance in the *Staphylococcaceae* have been reported^46,47^. However, these genes were not detected in *M. sciuri* isolates. We postulate that the phenotypic resistance exhibited by these isolates could be through various alternative mechanisms or resistance determinants. These observations were also reported in mammaliicoccal isolates obtained from German dairy farms^35^.

The *M. sciuri* multi-locus sequencing typing scheme (https://pubmlst.org/organisms/mammaliicoccus-sciuri) was established in 2020, and various STs have been described in Austria, Canada, Thailand, India, Switzerland, and some African countries. Two new STs (ST233 and ST234) were identified in the *M. sciuri* isolates from *hipposideros* bats. Based on the NJT constructed from the concatenated sequences of the STs on the database (Fig. 2), they did not share any similar alleles with previous STs reported in Nigeria. However, they clustered (with five to six similar alleles) among isolates with STs from different animals in Canada, Switzerland, India, and Thailand (Fig. 2 and Supplementary Table 2). In addition, some isolates in these STs cause mastitis in animals. The virulence and zoonotic potential of isolates in these STs are still unknown. Therefore, there is a need for surveillance and monitoring of *M. sciuri* in Nigeria to catalogue and elucidate their role in antibiotic drug resistance.

Our study has some limitations. Firstly, we were unable to capture many bats within the study period thereby affecting the number of isolates obtained. This could affect the accurate number and diversity of non-aureus staphylococci present in the studied insectivorous bat. Secondly, the study design could not investigate other components in the “One-Health” concept; the environment and humans, coexisting with these bats. Hence, the study could not ascertain NAS and M. sciuri, and the dissemination of their antibiotic resistance gene determinants in the human population and the environment.

## Conclusion

This study described the molecular characterization, antibiotic resistance and virulence determinants in non-aureus staphylococci and *M. sciuri* colonizing *Hipposiderous* bats. This further highlights the need to encourage the inclusion of wild animals in the One-Health approach as they may serve as reservoirs of potential pathogens that could cause zoonotic infections and the transfer of antibiotic-resistance genes. Some antibiotic resistance determinants have not been captured in the antibiotic resistance database as variability has been reported to be high among antibiotic resistance genes within the family *Staphylococcaceae*. Therefore, extensive and well-curated antibiotic resistance gene databases may be required to monitor and investigate antibiotic resistance in the family *Staphylococcaceae*.

## Methods

### Animal sampling

The bats were captured using a mist net at the basement of the Centre for Energy Research and Development building, Obafemi Awolowo University, Ile-Ife, Nigeria. Pharyngeal swabs were obtained using sterile cotton swabs moistened with normal saline. Morphometric identification of bats was performed, and the forearm length, sex, species, reproductive status, and body mass. Species identification of the bats was confirmed by DNA barcoding of the mitochondrial cytochrome b (MT-Cytb) genes, as previously reported^48,49^. The sampling period was from December 2020 to January 2021.

### Bacterial isolation and identification

The pharyngeal swabs were inoculated in 5 ml sterile nutrient broth (Merck, Darmstadt, Germany) and incubated overnight at 37 °C. The bacterial culture was streaked on mannitol salt agar (MAST diagnostics, UK) and incubated at 37 °C for 48 h. Colonies with staphylococci-like morphological characteristics (yellow/cream, round, convex, entire) were selected for phenotypic identification based on Gram stain reaction, catalase, coagulase, DNase (positive results), and oxidase (negative results) tests. PCR amplification of the elongation factor (*tuf*) gene^50^ was performed on the staphylococcal isolates (Supplementary Table 3). The identity of the isolates was confirmed using the MALDI-TOF MS (Bruker Daltonic, Germany).

### Antibiotic susceptibility testing

Antibiotic susceptibility testing was conducted using the disc diffusion technique. The antibiotics included cefoxitin (30 µg), chloramphenicol (30 µg), clindamycin (2 µg), ciprofloxacin (5 µg), erythromycin (15 µg), fusidic acid (10 µg), and gentamicin (10 µg). Others include mupirocin (5 µg and 200 µg), penicillin G (10U), rifampicin (5 µg), tetracycline (30 µg), trimethoprim (5 µg), and trimethoprim-sulfamethoxazole (1.25/23.75 µg). The zone of inhibition was measured and interpreted using the guidelines of the Clinical and Laboratory Standard Institute^51^. The guidelines for the British Standard for Antimicrobial Chemotherapy^52^ were applied to determine susceptibility to fusidic acid and mupirocin.

### Whole genome sequencing and analysis

All the isolates were subcultured on blood agar. DNA extraction was performed using the Qiagen DNeasy Blood and Tissue kit (Qiagen, USA) with an elution volume of 100 μl.

DNA samples were quantified using a Qubit fluorometer (ThermoFisher Scientific) based on dsDNA High sensitivity assay. Sequencing libraries were prepared using the Nextera DNA flex preparation kit (Illumina, USA). Library preparation was adopted from the Standard Operating Procedure of the United States CDC PulseNet Nextera DNA Flex^53^, and the samples were sequenced using the Miseq Illumina platform at the African Center of Excellence for Genomics of Infectious Diseases (ACEGID) Redeemer’s University, Nigeria.

FASTQ paired-end reads were processed with fastp v0.20.1 to improve the quality of the reads generated from the Illumina MiSeq. The processed reads were further analyzed with centrifuge v1.0.4 to validate the isolates and check for possible contamination. De novo assembly was performed with SPADES v3.13.0 on processed FASTQ files to generate contigs for each isolate. The quality of the contigs was assessed with Quast v5.0.2. AMR gene identification was performed with abricate v1.0.1 using CARD and ResFinder (https://cge.food.dtu.dk/services/ResFinder-4.1/- accessed 4 June 2023) and Plasmidfinder databases. SCC*mec* elements were investigated using the SCC*mec*Finder 1.2 (https://cge.food.dtu.dk/services/SCCmecFinder/- accessed 4 June 2023). Furthermore, virulence factors were detected using the VFanalyzer of the Virulence Factor Database (VFDB) using the default settings^54^. All the genome assemblies of *M. fleurettii*, *M. lentus*, *M. sciuri, M. vitulinus S. gallinarum* and *S. nepalensis* from NCBI were retrieved with contig assembly level and excluding atypical genomes used as selection criteria, yielding a total of 449 assemblies (sequence length ranging from 2.2 Mbp to 3.4 Mbp). The contigs and those generated in this study were annotated with Prokka^55^ and a pan-genome analysis was carried out with Roary^56^ to generate a core genome alignment and further analyzed using IQTREE^57^ to construct a maximum likelihood phylogenetic tree with an ultrafast bootstrap value of 1000 and general time reversible (GTR) model. SNP distances were calculated from the core genome alignment using snp-dists (https://github.com/tseemann/snp-dists).

### Multi-locus sequence typing (MLST)

MLST was performed on the *M. sciuri* isolates as previously described^58^. The sequence types (STs) were determined according to the MLST database (http://www.pubmslt.org). A NJ tree was constructed using the concatenated nucleotide sequences from the MLST database and visualized using the interactive Tree of Life (iTOL) tool^59^.

### Ethics statement

The ARRIVE (Animal Research: Reporting of In Vivo Experiments) guidelines' recommendations were followed in this study's performance and reporting of the experiments especially during sample collection. Ethical approval for the study was obtained from the Animal Care and Use Committee of the National Veterinary Research Institute (NVRI), Vom, Nigeria (approval number AEC/03/65/19). Approval was also obtained from the management of Obafemi Awolowo University, Ile-Ife, Nigeria prior to sample collection. All the samples were collected under the veterinarian’s supervision in full compliance with the local ethical and legal guidelines, and bats were immediately released after sampling.

### Supplementary Information

Supplementary Information 1.

Supplementary Information 2.

Supplementary Information 3.

## Data Availability

The sequencing raw reads for this study and the generated genomes have been deposited in NCBI under the BioProject accession number PRJNA905385.
